# Supplementary material for: Time-series transcriptome analysis identified differentially expressed genes in broiler chicken infected with mixed Eimeria species
Source: Front Genet. 2022 Aug 8;13:886781. doi: 10.3389/fgene.2022.886781 (PMC9393255; doi:10.3389/fgene.2022.886781)
Supplement: Supplementary file 2 [file DataSheet1.ZIP › 4dpi_KEGG.Gsea.1625100298584/KEGG_FOLATE_BIOSYNTHESIS.html]

Details for gene set KEGG\_FOLATE\_BIOSYNTHESIS[GSEA]

|  || Dataset | TMM\_4dpi\_gct\_format\_4dpi\_gct\_format.Class\_4dpi.cls #PC\_versus\_NC.Class\_4dpi.cls #PC\_versus\_NC\_repos |
| Phenotype | Class\_4dpi.cls#PC\_versus\_NC\_repos |
| Upregulated in class | 1 |
| GeneSet | KEGG\_FOLATE\_BIOSYNTHESIS |
| Enrichment Score (ES) | 0.57464033 |
| Normalized Enrichment Score (NES) | 1.3247486 |
| Nominal p-value | 0.15963513 |
| FDR q-value | 0.5686237 |
| FWER p-Value | 1.0 |
Table: GSEA Results Summary

  

Fig 1: Enrichment plot: KEGG\_FOLATE\_BIOSYNTHESIS      
 Profile of the Running ES Score & Positions of GeneSet Members on the Rank Ordered List

  

| SYMBOL | TITLE | RANK IN GENE LIST | RANK METRIC SCORE | RUNNING ES | CORE ENRICHMENT || 1 | GCH1 | na | 144 | 1.480 | 0.4275 | Yes |
| 2 | GGH | na | 1884 | 0.442 | 0.4137 | Yes |
| 3 | ALPP | na | 2681 | 0.326 | 0.4442 | Yes |
| 4 | DHFR | na | 3066 | 0.272 | 0.4929 | Yes |
| 5 | ALPL | na | 3097 | 0.269 | 0.5703 | Yes |
| 6 | PTS | na | 3739 | 0.194 | 0.5746 | Yes |
| 7 | FPGS | na | 5396 | 0.046 | 0.4503 | No |
| 8 | QDPR | na | 7495 | -0.135 | 0.3155 | No |
| 9 | SPR | na | 8252 | -0.204 | 0.3131 | No |
Table: GSEA details [plain text format]

  

Fig 2: KEGG\_FOLATE\_BIOSYNTHESIS      
 Blue-Pink O' Gram in the Space of the Analyzed GeneSet

  

Fig 3: KEGG\_FOLATE\_BIOSYNTHESIS: Random ES distribution      
 Gene set null distribution of ES for **KEGG\_FOLATE\_BIOSYNTHESIS**
